# Solvent-Free and Cost-Efficient Fabrication of a High-Performance Nanocomposite Sensor for Recording of Electrophysiological Signals

**DOI:** 10.3390/bios14040188

**Published:** 2024-04-11

**Authors:** Shuyun Zhuo, Anan Zhang, Alexandre Tessier, Chris Williams, Shideh Kabiri Ameri

**Affiliations:** 1 Department of Electrical and Computer Engineering, Queen’s University, Kingston, ON K7L 3N6, Canada; 2 Centre for Neuroscience Studies, Queen’s University, Kingston, ON K7L 3N6, Canada

**Keywords:** solvent free, compression, nanocomposite sensor, electrophysiological, skin impedance, signal-to-noise ratio

## Abstract

Carbon nanotube (CNT)-based nanocomposites have found applications in making sensors for various types of physiological sensing. However, the sensors’ fabrication process is usually complex, multistep, and requires longtime mixing and hazardous solvents that can be harmful to the environment. Here, we report a flexible dry silver (Ag)/CNT/polydimethylsiloxane (PDMS) nanocomposite-based sensor made by a solvent-free, low-temperature, time-effective, and simple approach for electrophysiological recording. By mechanical compression and thermal treatment of Ag/CNT, a connected conductive network of the fillers was formed, after which the PDMS was added as a polymer matrix. The CNTs make a continuous network for electrons transport, endowing the nanocomposite with high electrical conductivity, mechanical strength, and durability. This process is solvent-free and does not require a high temperature or complex mixing procedure. The sensor shows high flexibility and good conductivity. High-quality electroencephalography (EEG) and electrooculography (EOG) were performed using fabricated dry sensors. Our results show that the Ag/CNT/PDMS sensor has comparable skin–sensor interface impedance with commercial Ag/AgCl-coated dry electrodes, better performance for noninvasive electrophysiological signal recording, and a higher signal-to-noise ratio (SNR) even after 8 months of storage. The SNR of electrophysiological signal recording was measured to be 26.83 dB for our developed sensors versus 25.23 dB for commercial Ag/AgCl-coated dry electrodes. Our process of compress-heating the functional fillers provides a universal approach to fabricate various types of nanocomposites with different nanofillers and desired electrical and mechanical properties.

## 1. Introduction

The development of soft and flexible wearable sensors has attracted attention due to the mechanical compatibility of such sensors with soft and stretchable tissue, electrical conductivity, and biocompatibility. Such wearable sensors, when integrated with electronic circuits, provide a promising platform for human–machine interface, rehabilitation, medical diagnosis, and continuous health monitoring [1,2,3,4,5,6,7,8]. To create a conformable and robust interface between the sensor and soft, dynamic human skin, various approaches have been implemented, including exploiting deformable structures and employing intrinsically flexible materials [9,10,11,12,13,14,15]. During recent decades, various types of soft and conductive polymers and composites have been developed. These composites and polymers are made of polymers and/or elastomers with conductive fillers, such as carbon-based nanomaterials and metal nanoparticles [16,17,18,19,20,21,22]. Among the developed composites, carbon nanotube (CNT)-based nanocomposites have been widely used for the fabrication of wearable electronics due to the inherent stretchability, high electrical conductivity, and processibility of CNTs as conductive fillers in many polymer networks [23,24,25,26,27]. The conventional strategies used to fabricate CNT/polymer nanocomposites are solution mixing and dry/melt blending [20,24,28,29]. The solution mixing method is technically simple and scalable, but it requires the consumption of a large amount of solvent, which is harmful to the environment [30,31]. Melt blending is toxin-free and cost-effective, but it is only compatible with thermoplastic polymers. Also, poor dispersion of the filler in the polymer matrix specifically with a higher filler content is a likely scenario that affects the electrical conductivity and mechanical characteristics of nanocomposites and increases the polymer’s stiffness [32]. Thermoplastic-based nanocomposites are also made by dry mixing CNT and polymer powders followed by hot pressing, but the high degree of hardness of polymers limits their application in flexible devices [33]. Unconventional methods of making CNT/polymer nanocomposites include the spray coating and drop drying of CNT dispersion on the surface of polymer fibers or foams, though they may not be safe to use as biosensors mainly because of the poor adhesion of CNT to the polymer or foam surfaces [34,35,36,37]. The use of CVD-grown CNTs to make conductive nanocomposites with anisotropic strength has been reported as well, but only thin layers of conductive nanocomposites can be made by coating the grown CNTs with a polymer precursor, and the fabrication process is complex [38,39]. To address the abovementioned issues, we have developed a low-cost, time-effective, scalable, solvent-free method to fabricate CNT nanomaterials with high electrical conductivity.

To form a connected network of CNTs in the polydimethylsiloxane (PDMS) matrix and ensure good electrical conductivity, CNTs were compressed and thermally treated before soaking them in PDMS and curing the PDMS. The CNTs form a stable network under compression, which results in the high conductivity of nanocomposites and their enhanced mechanical strength and durability. This process (1) addresses the use of a large amount of chemicals and solvents and high temperatures in the conventional methods of making nanocomposites, (2) suggests a universal approach to fabricating various types of nanocomposites with different fillers and desired electrical and mechanical properties, and (3) enables one-step simple and combined surface modification with silver unlike conventional methods of surface modification that are multistep, complex, and require further chemical processing and energy consumption. Sensors produced using our developed method are reusable, have a large life span, and show robust operation; further, they do not cause skin irritations and allergic reactions.

## 2. Results and Discussions

**Figure 1:**
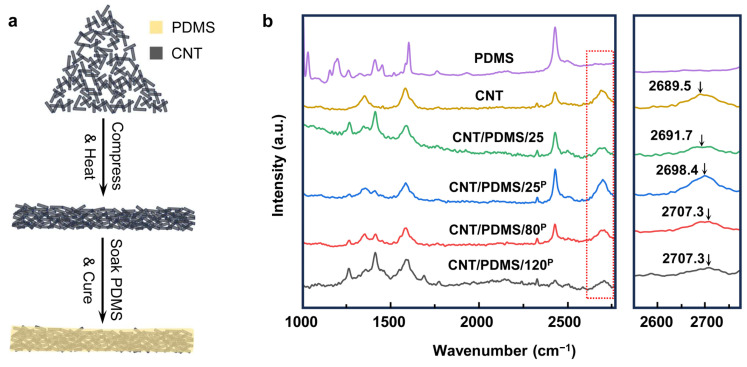
(**a**) Illustration of the fabrication process of compressed CNT/PDMS nanocomposites. (**b**) Raman spectrum of PDMS, CNT, CNT/PDMS/25, CNT/PDMS/25^P^, CNT/PDMS/80^P^, and CNT/PDMS/120^P^.

The solvent-free and cost-efficient process of making CNT/PDMS nanocomposites is shown in Figure 1a. PDMS was selected as the polymer matrix due to its nontoxicity, flexibility, thermal stability, simple processing, and biocompatibility [40,41]. To fabricate CNT/PDMS nanocomposite-based sensors containing a thin layer of silver nanoparticles at the interface with the skin, first the electrical and mechanical characteristics of CNT/PDMS nanocomposites were studied, and our solvent-free fabrication process was optimized.

To fabricate CNT/PDMS nanocomposites-based sensors, a continuous and connected network of CNTs over a large area was formed by co-applying compression and heat to the CNTs to create an electrically conductive network. Then, the CNT network was soaked in the PDMS precursor, and the PDMS was cured to form flexible electrodes, as shown in Figure 1a and Figure S1. At first, the effects of the compression and heat applied to CNTs on the electrical and mechanical characteristics of nanocomposite samples were studied to find the optimum fabrication parameters for making the sensor. A series of CNT/PDMS nanocomposites were prepared with/without applying compression at different temperatures. CNT/PDMS/25 was fabricated at 25 °C without compressing the CNTs; CNT/PDMS/25^P^, CNT/PDMS/80^P^, and CNT/PDMS/120^P^ were made by compressing the CNTs under 7.4 × 10^3^ N/m^2^ of pressure at 25, 80, and 120 °C, respectively. Figure 1b shows the results of Raman spectroscopy performed on the CNT/PDMS nanocomposites made at different compressions and temperatures. Our results suggest that the applied external compression/heat to CNTs and the presence of the polymer matrix induce residual strain on the CNTs, resulting in shifting the characteristic Raman peak of CNTs from 2689.5 cm^−1^ to higher wavenumbers [42,43,44]. As demonstrated in Figure 1b, when no compression and heat is applied to CNTs, the Raman peak of CNT/PDMS/25 appears at 2691.7 cm^−1^, while the Raman peak of CNT/PDMS/25^P^ fabricated with compression is located at 2698.4 cm^−1^, and it further shifted to 2707.3 cm^−1^ when the CNTs were compressed under the same pressure at higher temperatures of 80 °C (CNT/PDMS/80^P^) and 120 °C (CNT/PDMS/120^P^). These results show that both heat and compression cause a rise in the residual strain in CNTs and contribute to forming a more compact CNT network, therefore enhancing the electrical and mechanical characteristics of the CNT/PDMS nanocomposites.

**Figure 2:**
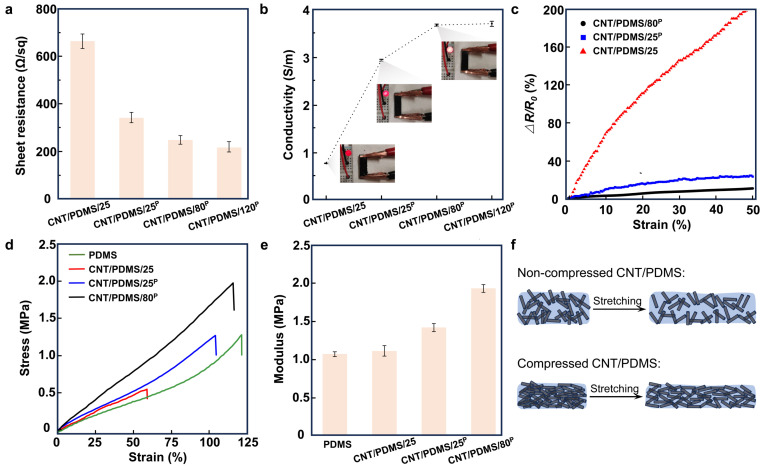
The effect of applying compression and heat during the fabrication of CNT/PDMS nanocomposite on its electrical and mechanical properties. (**a**) The sheet resistance and (**b**) the conductivity of CNT/PDMS/25, CNT/PDMS/25^P^, CNT/PDMS/80^P^, and CNT/PDMS/120^P^ samples; inset photos of b show the brightness of an LED light connected in series to nanocomposite samples. (**c**) The changes in the resistance of CNT/PDMS/25, CNT/PDMS/25^P^, and CNT/PDMS/80^P^ samples versus tensile strain. (**d**) The stress–strain curves of PDMS, CNT/PDMS/25, CNT/PDMS/25^P^, and CNT/PDMS/80^P^ nanocomposites. (**e**) The Young’s modulus of PDMS, CNT/PDMS/25, CNT/PDMS/25^P^, and CNT/PDMS/80^P^ nanocomposites. (**f**) Illustration of the changes induced in the network of CNTs in nanocomposites due to applying tensile strain to them.

The sheet resistances of CNT/PDMS/25, CNT/PDMS/25^P^, CNT/PDMS/80^P^, and CNT/PDMS/120^P^ were measured to be 663 ± 32, 340 ± 24, 247 ± 19, and 216 ± 25 Ω/sq, respectively (Figure 2a). The results indicate that the conductivity of the nanocomposite increases by increasing the temperature from 25 °C to 120 °C and by applying compression to the CNTs, as shown in Figure 2b. The conductivity was 0.75 ± 0.01, 2.93 ± 0.02, 3.67 ± 0.02, and 3.70 ± 0.05 S/m for CNT/PDMS/25, CNT/PDMS/25^P^, CNT/PDMS/80^P^, and CNT/PDMS/120^P^, respectively. Figure 2b indicates a 389% improvement in the conductivity of CNT/PDMS/80^P^ in comparison with CNT/PDMS/25. However, our results show that further increasing the temperature from 80 to 120 °C did not improve the conductivity of the nanocomposites significantly, and therefore CNT/PDMS/80^P^ nanocomposite is the best candidate for the fabrication of the sensors. Figure 2c shows the effect of mechanical tensile strain on the electrical resistance of nanocomposites produced at different conditions. The results show that the CNT/PDMS/25 nanocomposite is the most susceptible sample to the strain, showing a 200% increase in the electrical resistance due to applying 50% of tensile strain, while the nanocomposite produced using compressed CNTs at 80 °C shows only a 10% increase in the electrical resistance when 50% of tensile strain was applied. This is mainly due to the better-formed network of CNTs in the CNT/PDMS/80^P^ nanocomposite.

Figure 2d shows the stress–strain plot of PDMS, CNT/PDMS/25, CNT/PDMS/25^P^, and CNT/PDMS/80^P^. As the results indicate, the nanocomposite made under no compression at 25 °C shows poor mechanical performance with a much lower elongation at break of 55%, in comparison with the pure PDMS matrix with an elongation at break of approximately 120%. This can be attributed to the formation of voids and pores in the CNT/PDMS/25 sample (Supplementary Figure S2). The results show that the elongation at break increases by applying compression and heat to CNTs. The CNT/PDMS nanocomposites fabricated by compressing CNTs at 25 and 80 °C show improved elongation at break of 100% and 110%, respectively, of which the latter is close to the PDMS matrix. The extracted values of the Young’s modulus of the samples, shown in Figure 2e, suggest moduli of 1.06, 1.10, 1.41, and 1.92 MPa for PDMS, CNT/PDMS/25, CNT/PDMS/25^P^, and CNT/PDMS/80^P^, respectively. These results show that the incorporation of CNTs has greatly improved the mechanical strength of the compressed CNT/PDMS nanocomposites, which is mainly attributed to the formation of CNT networks, as demonstrated in Figure 2f.

The collective electrical and mechanical characteristics of our studied CNT/PDMS nanocomposites produced under different applied compressions and temperatures suggest that the compressed CNT/PDMS nanocomposite prepared under 7.4 × 10^3^ N/m^2^ pressure at 80 °C (CNT/PDMS/80^P^) shows higher electrical conductivity as well as larger elongation at break and a higher elastic modulus, indicating the mechanical strength and durability of the developed nanocomposites.

**Figure 3:**
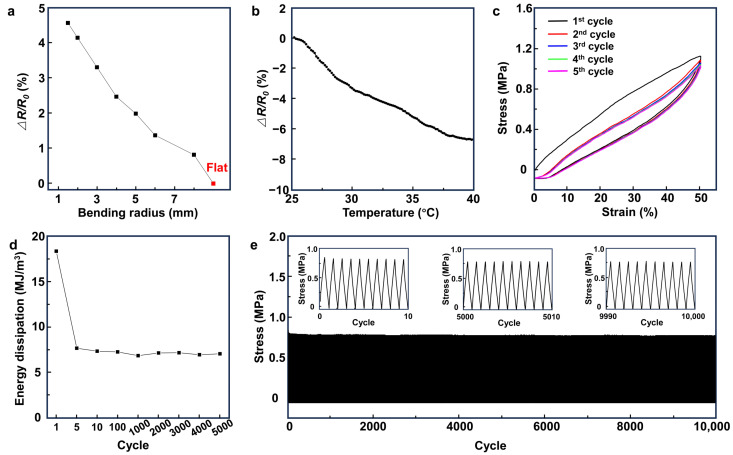
The electrical and mechanical stability and durability of the CNT/PDMS nanocomposites. The change in the electrical resistance of CNT/PDMS/80^P^ versus (**a**) bending radius and (**b**) temperature. (**c**) The loading–unloading curves (hysteresis) of successive cycles of CNT/PDMS/80^P^ sample at a strain of 50%. (**d**) The energy dissipation at 1, 5, 10, 100, 1000, 2000, 3000, 4000, and 5000 cycles at a strain of 50%. (**e**) A total of 10,000 strain cycles of 30% for CNT/PDMS/80^P^ sample.

Figure 3a and Figure S3 show the change in the electrical resistance of the CNT/PDMS/80^P^ nanocomposites versus the bending radius. Our results indicate the high stability of the nanocomposite polymer, with only a 4.5% increase in the electrical resistance at a 1.5 mm bending radius. The changes in the electrical resistance of the CNT/PDMS/80^P^ nanocomposites within the temperature range of 25 °C to 40 °C, presented in Figure 3b, show only a 6% decrease in the electrical resistance of the nanocomposite, indicating that the change in the skin’s temperature would not have an adverse effect on the electrophysiological signal measurements. The hysteresis plot in Figure 3c shows five stress–strain cycles of CNT/PDMS/80^P^ nanocomposites. The CNT/PDMS/80^P^ nanocomposite shows a closed hysteresis loop after the first stress–strain cycle when the tensile strain applied to the nanocomposite changes from 0% to 50% and from 50% to 0%, suggesting the good elasticity of the nanocomposite. To investigate the fatigue resistance of CNT/PDMS/80^P^ nanocomposites, 10,000 and 5000 successive cycles of tensile loading–unloading were carried out for up to 30% and 50% of applied tensile strain, respectively (Figure 3d,e and Figure S4). The measured stress reduced by only 0.08 and 0.26 MPa after 10,000 strain cycles of 30% and 5000 successive cycles of 50% tensile strain. The corresponding energy dissipation in the sample under 50% of applied tensile strain shows to be stable around 7.5 MJ/m^3^ within the first five cycles, indicating the good anti-fatigue resistance and elasticity of the CNT/PDMS/80^P^ nanocomposites.

**Figure 4:**
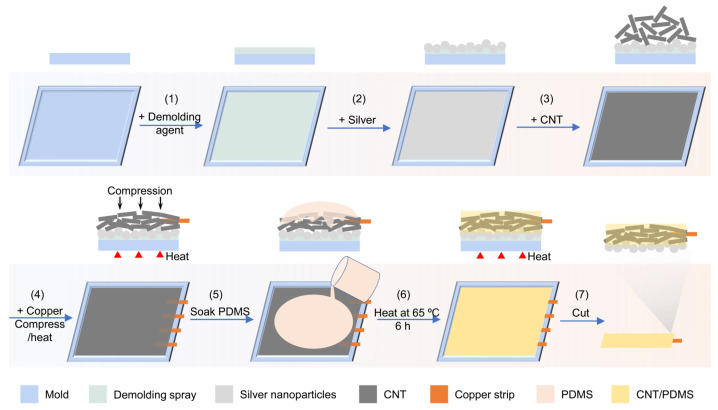
Schematic of the fabrication process of silver nanoparticle/CNT/PDMS sensors. After applying the demolding spray on the surface of the mold, a thin layer of silver nanoparticles was added into the mold, followed by filling the mold with CNT, placing a copper strip, compressing, and applying heat. By soaking this combination in PDMS and curing the PDMS, the fabrication of the sensor was completed.

To fabricate the sensor, in the first step, an acrylonitrile butadiene styrene (ABS) mold was printed using a 3D printer (Figure 4). Then, the surface of the mold was uniformly coated with silver nanoparticles to improve the skin–sensor interface before adding CNTs into the mold and soaking the network in PDMS. Next, multi-walled carbon nanotubes with a length and diameter of 5~20 µm and 16 ± 3.6 nm, respectively, were used to fill the mold. To make secure connections to the circuits, a thin copper strip (with a thickness of 220 μm) was placed within the CNT powder, and then they were compressed under 7.4 × 10^3^ N/m^2^ pressure at 80 °C for 6 h. Compression results in forming a network of CNTs [33,45]. In the next step, liquid PDMS (base and curing agent with a weight ratio of 10:1) was poured into the mold containing CNTs and silver nanoparticles. The liquid PDMS precursor penetrates within the formed network of CNT in 30 min. Finally, the sample was heated at 65 °C for 6 h to cure the PDMS. The sensor was then de-molded and used for electrophysiological recording. Our strategy of compressing and heating the conductive fillers provides a universal approach to fabricate conductive materials in a solvent-free and energy-saving way, compared with solution mixing and dry/melt blending that require a large amount of solvent and high energy consumption (such as a long milling times [24,28] and high temperature [29]), respectively.

**Figure 5:**
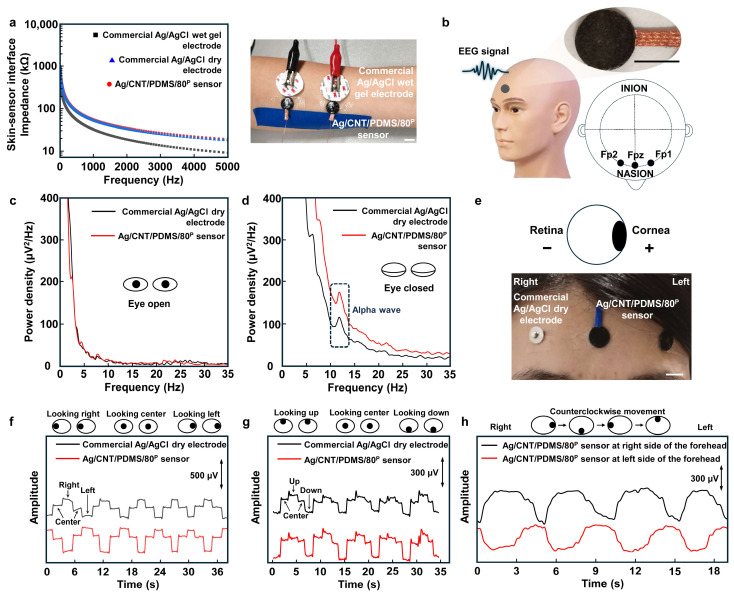
The sensing performance of the Ag/CNT/PDMS/80^P^ sensor. (**a**) The commercial Ag/AgCl dry electrode–, Ag/AgCl wet gel electrode– and Ag/CNT/PDMS/80^P^ sensor–skin interface impedances measured on the forearm. The photo shows the measurement setup; scale bar is 1 cm. (**b**) The illustration of recording EEG signals; inset shows the photo of an Ag/CNT/PDMS/80^P^ sensor. The scale bar is 1 cm. The comparison of EEG signals detected when the eyes were (**c**) open and (**d**) closed. (**e**) The configuration of the sensors for EOG measurements using commercial Ag/AgCl dry electrode and the Ag/CNT/PDMS/80^P^ sensor. The scale bar is 1 cm. EOG signals recorded during eye movements of (**f**) looking left and right, (**g**) looking up and down, and (**h**) counterclockwise.

To study the suitability of the fabricated sensor for electrophysiological recording, two sensors were placed on the forearm next to the commercial Ag/AgCl wet gel electrodes and commercial Ag/AgCl dry electrodes as gold standards, and the electrode–skin interface impedance in the frequency range of 20 Hz to 5 kHz was measured using all these three types of sensors and electrodes. The results shown in Figure 5a indicate that the sensor–skin interface impedance of the Ag/CNT/PDMS/80^P^ sensor is similar to that of the commercial Ag/AgCl dry electrodes and is slightly higher than Ag/AgCl wet gel electrodes (it is known that Ag/AgCl wet gel electrodes have the lowest electrode–skin interface impedance of all types of electrodes). These results indicate that our sensor makes an effective interface with the skin, which is critical for recording various types of electrophysiological signals. The application of our sensors for electrophysiological recordings was demonstrated by performing electroencephalography (EEG) and electrooculography (EOG) on the forehead. No conductive gel was applied at the interface between the sensors and the skin (Figure 5b). Figure 5c,d show the recoded alpha rhythm at 8–13 Hz by the Ag/CNT/PDMS/80^P^ sensor and Ag/AgCl dry electrode at the Fp1 and Fp2 positions on the forehead when the eyes are open and when they are closed [46,47,48]. The Ag/CNT/PDMS/80^P^ sensor was placed in the Fpz position as the reference and ground electrodes [49,50]. The alpha rhythm was expected to appear when the eyes were closed and the subject was relaxing. EEG signals were measured while the subject kept their eyes open for 1 min and then closed them for another 1 min. The fast Fourier transform (FFT) results of the recorded data in Figure 5c,d show a clear alpha signal with a peak frequency of 12 Hz when the eyes were closed. It is worth noting that the amplitude of the alpha signal detected by the Ag/CNT/PDMS/80^P^ sensor is higher than that measured by the commercial dry Ag/AgCl electrode. This is due to two main reasons: (1) higher electrical conductivity of Ag/CNT/PDMS/80^P^ because of forming a compact network of CNTs in PDMS, and (2) improved interface with the skin due to the lower mechanical stiffness of our sensor.

The application of the sensor for EOG recording was demonstrated as well. The EOG measures the electrostatic potential that exists between the retina and cornea during eyes movements [51,52]. Figure 5e shows the placement of our developed sensors and commercial dry Ag/AgCl electrodes as a standard electrode on the forehead for EOG recordings, where two Ag/CNT/PDMS/80^P^ sensors are used as working and reference/ground electrodes, respectively, due to their high flexibility and conductivity [53,54,55]. The EOG data recorded by the sensors were wirelessly transmitted to a laptop for signal processing. Figure 5f,g show the EOG signals recorded during ‘left’-, ‘right’-, ‘up’-, and ‘down’-ward movements of the eyes. Clear EOG signals were recorded using both the commercial Ag/AgCl dry electrodes and Ag/CNT/PDMS/80^P^ sensors. The results show that the signal recorded using our sensor has a higher signal-to-noise ratio (SNR) of 26.83 dB compared with 25.23 dB for the commercial electrode. Figure 5h shows the EOG signals recorded using Ag/CNT/PDMS/80^P^ sensors while the subject moved their eyes counterclockwise. To record real-time EOG during the rolling of the eyes, three Ag/CNT/PDMS/80^P^ sensors were used, two attached above the eyebrows as working electrodes and one attached at the center of the forehead as a ground/reference electrode. The EOG signals in Figure 5h recorded by the sensor located on the right side of the forehead (black curve) and the sensor located on the left side of the forehead (red curve) during rolling of the eyes clearly show the recorded potential of the retina (negative) and cornea (positive) by each sensor while the eyes were moving. It is worth mentioning that during eye rolling, when both eyes are in their left corner, the cornea of the left eye that has positive potential is in the vicinity of the sensor on the left side of the forehead, and therefore we observe a positive potential recorded from the sensor on the left side of the forehead. However, at the same time, the retina of the right eyes with negative potential is in the vicinity of the sensor located on the right side of the forehead, and therefore we observe a negative potential recorded using the sensor located on the right side of the forehead. This results in a difference between the signals recorded using two sensors located on the left and right sides of the forehead. The EOG signals recorded during eye rolling could be further used for eye-movement-based communications, like eye writing.

**Figure 6:**
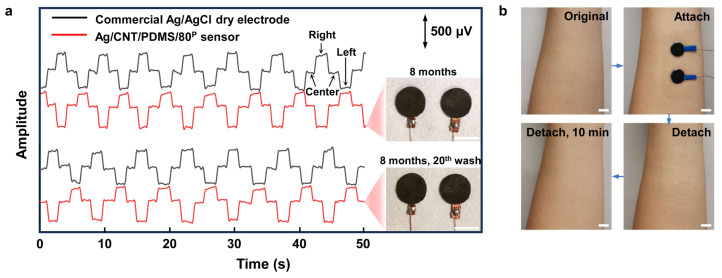
The sensing performance of the Ag/CNT/PDMS/80^P^ sensor on skin after 8 months of storage. (**a**) EOG signals recorded during the left–right eye movements measured by Ag/CNT/PDMS/80^P^ sensor that was used, washed, and cleaned 20 times and stored for 8 months. The inset photos show the Ag/CNT/PDMS/80^P^ sensors. (**b**) The skin before and after having the sensors on the forearm for 8 h. No redness or irritation were observed. The scale bar indicates 1 cm.

Good mechanical and electrical characteristics of the compressed CNT/PDMS nanocomposite resulted in high stability and durability of the Ag/CNT/PDMS/80^P^ sensors for long-term use. Figure 6a shows that high-quality EOG signals were recorded using Ag/CNT/PDMS/80^P^ sensors that were stored for 8 months. The SNR of the recorded EOG signals using this Ag/CNT/PDMS/80^P^ sensor was higher than that of the commercial Ag/AgCl dry electrodes. The high-quality recording and a higher SNR indicate that the electrical performance of Ag/CNT/PDMS/80^P^ sensors was not affected over time. Moreover, to demonstrate the reliability of our sensor for repeated daily use, we replicated the routine maintenance and reuse of our sensor by repeated rinsing of the Ag/CNT/PDMS/80^P^ sensor with water for 30s and letting dry in the ambient condition 20 times. As the inset photo of Figure 6a shows, there is no obvious change in the appearance of our sensors, and, further, the EOG measurements show no reduction in the SNR. Figure 6b shows that the Ag/CNT/PDMS/80^P^ sensor did not cause any irritation after 8 h of wearing it. Furthermore, the small size and light weight of our sensor make it a comfortable and user-friendly sensor for various applications in wearable technology.

## 3. Conclusions

In this work, CNT/PDMS nanocomposites were made by a solvent-free, cost-efficient, and facile fabrication process and characterized. A sensor for the electrophysiological recording was fabricated from a batch of nanocomposites in which the CNT network in the polymer matrix was formed under 7.4 × 10^3^ N/m^2^ of pressure at 80 °C. The sensor showed high electrical conductivity, mechanical strength, as well as durability and long-term reliability. The harmless and effective fabrication approach avoids the use of any solvents, chemical waste, and high temperatures. The resulting sensor showed the capability for high-quality electroencephalography (EEG) and electrooculography (EOG) recordings. The SNR of the recorded EOG signals using our sensors has been higher than that of commercial Ag/AgCl dry electrodes. In addition, the SNR of our sensors remains higher than that of the Ag/AgCl dry electrodes after being stored for 8 months and repeating 20 cycles of washing and drying, showing its potential applications for long-term use in wearable technology. Our developed fabrication method is efficient in making flexible sensors characterized by high conductivity, good durability, and a robust interface with the skin, which provides a universal strategy to produce various conductive materials utilizing different fillers.

## Data Availability

The original contributions presented in the study are included in the article/Supplementary Material, further inquiries can be directed to the corresponding author/s.

## Supplementary Materials

The following supporting information can be downloaded at: https://www.mdpi.com/article/10.3390/bios14040188/s1, Figure S1: Detailed schematic diagram of the fabrication process of compressed CNT/PDMS nanocomposites; Figure S2: Optical microscope images of (a), (b) the CNT/PDMS/80^P^ and (c), (d) CNT/PDMS/25; Figure S3: (a) Illustration of the process used to measure the change in electrical resistance of the sensor at different bending radii. (b) Photo showing the metal tubes with different radii used to bend the composites; Figure S4: Cyclic strain-stress test of the CNT/PDMS/80^P^ for 5000 cycles at 50% strain.
