# Supplementary material for: Solvent-Free and Cost-Efficient Fabrication of a High-Performance Nanocomposite Sensor for Recording of Electrophysiological Signals
Source: Biosensors (Basel). 2024 Apr 11;14(4):188. doi: 10.3390/bios14040188 (PMC11048393; doi:10.3390/bios14040188)
Supplement: Supplementary file 1 [file biosensors-14-00188-s001.zip › biosensors-2926869-supplementary.pdf]

biosensors

MDPI

Supplementary Materials

Solvent-Free and Cost-Efficient Fabrication of a High-Performance Nanocomposite Sensor for Recording of Electrophysiological Signals

Shuyun Zhuo [1], Anan Zhang [1], Alexandre Tessier [1], Chris Williams [1] and Shideh Kabiri Ameri [1,2,*]

[1] Department of Electrical and Computer Engineering, Queen's University, Kingston, ON K7L 3N6, Canada; shuyun.zhuo@queensu.ca (S.Z.); 17az20@queensu.ca (A.Z.); 19apt1@queensu.ca (A.T.); 12cmw7@queensu.ca (C.W.)
[2] Centre for Neuroscience Studies, Queen's University, Kingston, ON K7L 3N6, Canada
* Correspondence: shideh.ameri@queensu.ca

Characterizations:

Raman test was performed on PDMS, CNT, CNT/PDMS/25, CNT/PDMS/25^P, CNT/PDMS/80^P, and CNT/PDMS/120^P by using RENISHAW inVia Raman microscope with a wavenumber range of 1000-2800 cm^{-1}.

Optical microscope images were taken to characterize the CNT dispersion in PDMS through AmScope. The cross-sections of freshly made CNT/PDMS/25 and CNT/PDMS/80^P were studied.

Electrical property measurements: Sheet resistance was measured with an Eddycus TF lab 2020. To measure skin-sensor impedance, two sensors were attached 3 cm apart on the forearm with the help of 3M Tegaderm film and the impedance was measured using an LCR meter in the frequency range of 20 to 5000 Hz. Skin preparation was not done.

Mechanical tests: Strain-stress tests were conducted on PDMS, CNT/PDMS/25, CNT/PDMS/25^P and CNT/PDMS/80^P using a UniVert CellScale machine. Cyclic tests of CNT/PDMS/80^P samples were done at maximum strains of 50% for 5000 cycles and 30 % for 10000 cycles.

Electrophysiological recording: EOG and EEG signals were recorded with an OpenBCI board, and a digital 60 Hz notch filter was applied to the recorded data. Signal-to-noise ratio (SNR) was calculated using the following equation:

$$\text{SNR (dB)} = 20\ [\log_{10}\frac{A_{signal}}{A_{noise}}] \qquad \text{(S1)}$$

where A_{signal} and A_{noise} are the measured signal amplitude and noise amplitude, respectively. For durability test, the Ag/CNT/PDMS/80^{P} sensors were stored for 8 months at room temperature. The sensors were repeatedly washed by rinsing the surface with tap water for 30 s and dried in ambient atmosphere.

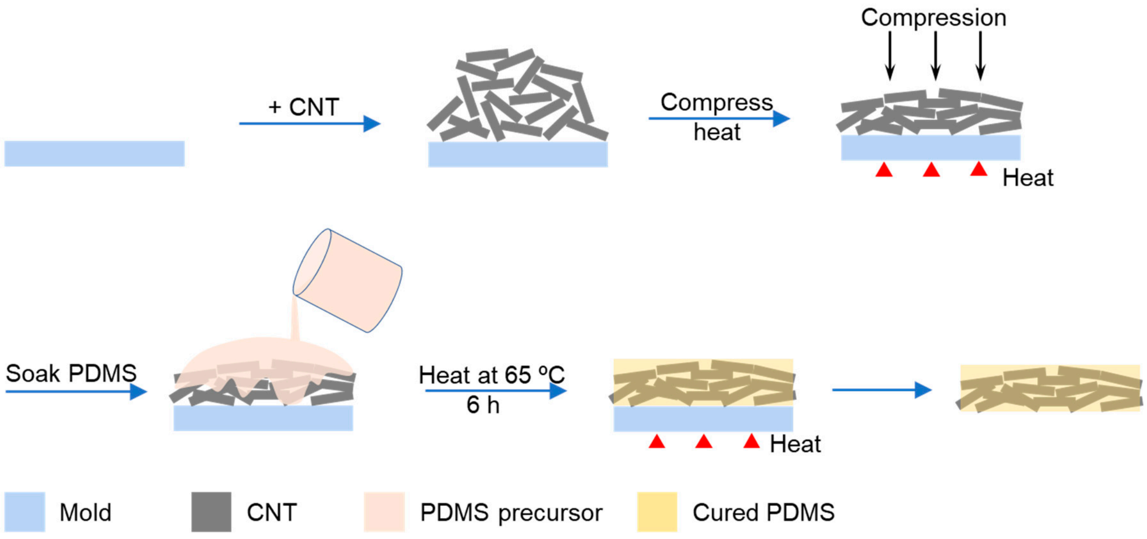

Figure S1. Detailed schematic diagram of the fabrication process of compressed CNT/PDMS nanocomposites.

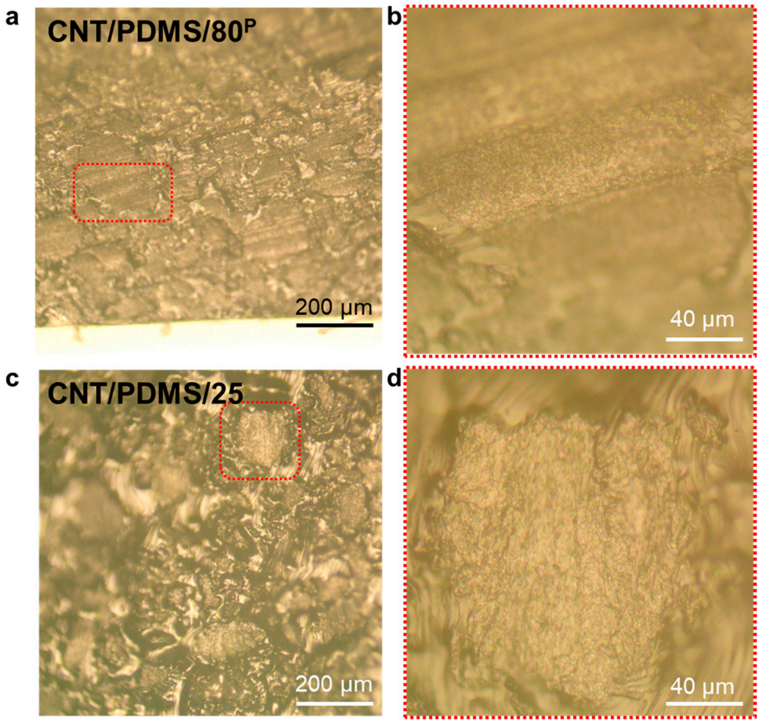

Figure S2. Optical microscope images of (a), (b) the CNT/PDMS/80^{P} and (c), (d) CNT/PDMS/25 .

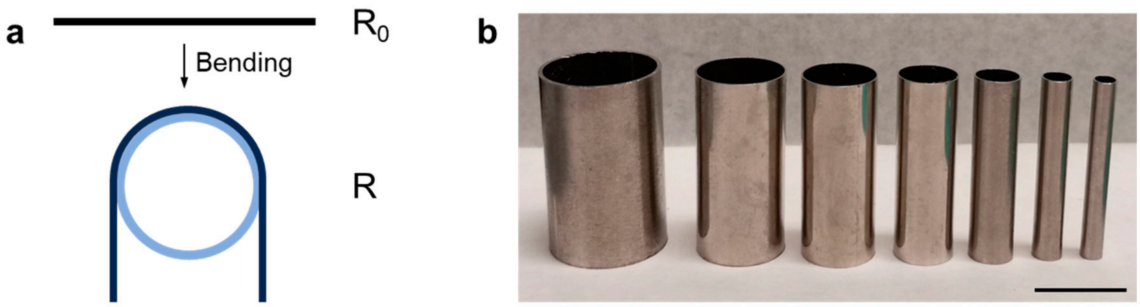

Figure S3. (a) Illustration of the process used to measure the change in electrical resistance of the sensor at different bending radii. (b) Photo showing the metal tubes with different radii used to bend the composites. Scale bar: 1 cm.

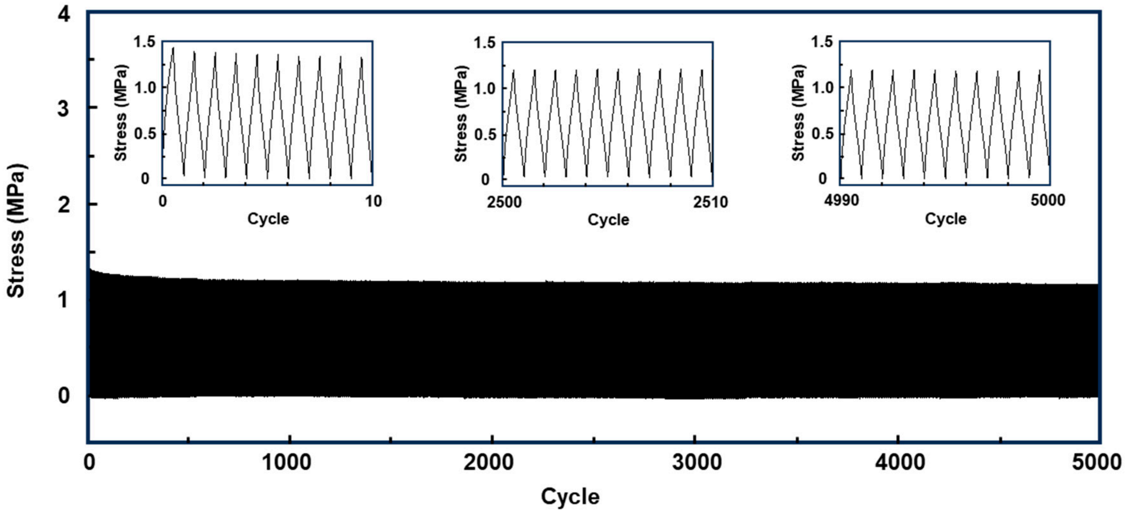

Figure S4. Cyclic strain-stress test of the CNT/PDMS/80[P] for 5000 cycles at 50% strain.
